# Lexical Bundles in Chemistry Research Articles

**DOI:** 10.3389/fpsyg.2022.906641

**Published:** 2022-05-25

**Authors:** Javad Zare, Leila Valipouri

**Affiliations:** ^1^Department of English Language and Literature, Kosar University of Bojnord, Bojnord, Iran; ^2^Department of English, University of Kashan, Kashan, Iran

**Keywords:** lexical bundle, discourse function, chemistry research article, abstract, introduction, results and discussion

## Abstract

The purpose of this corpus-based study was to investigate whether different sections in chemistry research articles, i.e., abstract, introduction, and results and discussion, rely on different sets of lexical bundles. Lexical bundles, associated with the above sections, were extracted from a corpus of 4 million words, comprising 1,185 chemistry research articles, using WordSmith Tools 5.0, and were categorized according to their functions. Altogether, 197 key bundles were identified in the three sections of chemistry research articles, 15 in the abstract, 99 in the introduction, and 83 in the results and discussion section. Two functions also emerged for lexical bundles in chemistry research articles, including purpose-oriented bundles, which refer to the aim/aims of the study; and literature-oriented bundles, which are used to refer to the literature. Altogether, the results showed that various sections in chemistry RAs are associated with specific sets of lexical bundles and, as such, deal with different rhetorical functions.

## Introduction

English for academic purposes (EAP) is concerned with teaching English to those who use it for study and research (Flowerdew and Peacock, 2001). Hyland (2006) defines EAP as “specialized English-language teaching grounded in the social, cognitive, and linguistic demands of academic target situations, providing focused instruction informed by an understanding of texts and the constraints of academic contexts” (p. 2). Our understanding of the features and constraints of different text types is the result of studies that have investigated different written and spoken genres. One of the features of written and spoken language use that has attracted the attention of many researchers is formulaic sequences, clusters, chunks, multi-word units, n-grams, or lexical bundles. Lexical bundles are “recurrent expressions regardless of their idiomaticity, and regardless of their structural status,” which “can be regarded as extended collocations; a bundle of words that show a statistical tendency to co-occur” in a register (Biber et al., 1999, p. 990). Research on lexical bundles shows that different genres, registers, and disciplines draw on particular types of bundles in their discourses (e.g., Biber et al., 1999; Cortes, 2002, 2004; Biber and Barbieri, 2007; Hyland, 2008a,b). This challenges the widely held assumption that there is a single core vocabulary, which can be equally useful for academic students in different fields of study (Hyland and Tse, 2007). Hyland (2008b) attributes similarities and differences between registers and disciplines in the use of lexical bundles to the purposes and audience for which different discourses are written. Therefore, further investigation needs to be conducted on the use of these multi-word sequences in and across different fields of study. In this respect, chemistry research articles (RAs) have been treated rather marginally in lexical bundles research. Furthermore, research suggests that different sections of research articles (RAs) differ from each other in their rhetorical structures and thus rely on different lexical and structural features (e.g., Swales, 1990; Lorés, 2004; Swales and Feak, 2010). As such, the use of lexical bundles might be associated with different sections of RAs.

In light of the above points and with a view to shedding light on the language of different sections of chemistry RAs, this study was an attempt to investigate the use of lexical bundles in different sections of chemistry Ras, drawing on a corpus-based discourse analytic approach. More specifically, we wanted to see if different sections in chemistry RAs, i.e., abstract, introduction, and results and discussion, rely on different sets of lexical bundles; if so, what these sequences are? In what ways they are structurally and functionally different? And how they are related to the specific functions of different RA sections? The findings of the study may inform theory in lexical bundles research across RA sections. The findings may also inform EAP practice by advising EAP learners, teachers, and material developers.

## Review of the Literature

### Lexical Bundles: Overview and Research

The term “lexical bundle” first appeared in the Longman Grammar of Spoken and Written English (LSWE) (Biber et al., 1999). Lexical bundles are fundamentally different from other kinds of formulaic expressions in three ways; First, lexical bundles are extremely common (Biber et al., 1999); second, they are not necessarily idiomatic in meaning (Conrad and Biber, 2005), that is, their meaning is retrievable from the meaning of its individual parts (e.g., *it is important to*); third, they do not typically represent a complete structural unit (Biber et al., 1999; Conrad and Biber, 2005). They are often phrasal (e.g., *in the case of*) or clausal (e.g., *I do not know what*) fragments with new fragments embedded (e.g., *is shown in figure*).

Lexical bundles have been investigated in different genres and registers in and across different disciplines both structurally and functionally (e.g., Biber and Conrad, 1999; Biber et al., 1999, 2004; Cortes, 2002, 2004; Conrad and Biber, 2005; Biber, 2006; Biber and Barbieri, 2007; Hyland, 2008a,b). The results of such studies show that (a) all university registers consist of lexical bundles but in different extents (Biber and Barbieri, 2007); bundles mostly consist of noun phrases (e.g., *the nature of the*), adjectival phrases (e.g., *is consistent with the*), prepositional phrases (e.g., *in the context of*), passive verbs (e.g., *is based on the*), and anticipatory (e.g., *it is possible that*) (Biber et al., 1999); most bundles in academic prose are parts of noun phrases and prepositional phrases (e.g., *the nature of the*) (Biber et al., 1999, 2004); across discipline, bundles in biology and history RAs are more phrasal than clausal (Cortes, 2002, 2004); functionally, both biology and history RAs have more referential bundles and text organizers than other types (Cortes, 2002, 2004).

Hyland (2008b) related similarities and differences between registers and disciplines in the use of lexical bundles to the purposes and audience for which different discourses are written. According to their purpose, lexical bundles are divided into different functional classifications. For example, Biber et al. (2004) divided the primary discourse functions of lexical bundles into stance expressing, discourse organizing, and referencing. “Stance bundles express attitudes or assessments that provide a frame for the interpretation of the following proposition” (e.g., *I do not know if*) (Conrad and Biber, 2005, p. 65). Discourse organizer bundles “reflect relationships between prior and coming discourse” (e.g., *let us have a look*) (Conrad and Biber, 2005, p. 67). “Referential bundles make direct reference to physical or abstract entities, or to the textual context” (e.g., *in the form of*) (Conrad and Biber, 2005, p. 67). On the other hand, Hyland (2008a) classified lexical bundles into three main categories of functions: research oriented, text oriented, and participant oriented. Research-oriented bundles help writers structure their activities and experiences. Text-oriented bundles deal with the organization of the text. Participant-oriented bundles deal with the reader or writer of the text. Research shows that structure and function of bundles are closely connected (Biber et al., 2004; Hyland, 2008a). For example, noun phrases with embedded *of* phrases are mostly research-oriented bundles, while prepositional phrases are, functionally, more text-oriented bundles (Hyland, 2008a).

### Research Article Subsections

Different sections in RAs, i.e., abstract, introduction, method, results and discussion, and conclusion, have been the focus of many genre analysis papers (e.g., Swales, 1990; Peacock, 2002; Yang and Allison, 2003; Koutsantoni, 2006; Bruce, 2009; Loi et al., 2016; Zare and Naseri, 2021a). The results of such investigations have generally shown that, in RAs, these sections follow different rhetorical structures or moves and thus rely on different sets of lexical or structural features. Gledhill (1995, 1996), for example, studied the use of word sequences in cancer RAs and found them diverse across different sections. Elsewhere, Lorés (2004) showed that abstracts “differ from RAs … in their function, in their rhetorical structure, and in their linguistic realizations” (p. 281). Function, rhetorical structure, and linguistic realizations, as noted further, are, “undoubtedly, closely connected: the function of an abstract will determine both its global structure and its linguistic realization” (Lorés, 2004, p. 281).

Abstracts perform different rhetorical functions, i.e., as “stand-alone mini-texts, giving readers a quick summary of a study’s topic, methodology, and findings”; as “screening devices, enabling the reader to decide whether to read the article as a whole”; as “previews, creating an interpretive frame that can guide reading”; and as “aids to indexing by professional indexers for large database services” (Huckin, 2001, p. 93). Hyland (2000) classified rhetorical moves of abstracts into five groups, namely introduction, purpose, method, product, and conclusion. Zare and Naseri (2021a) also found five moves, i.e. (1) establishing the territory or area of study, (2) identifying the problem, (3) introducing the present research, (4) organizing the paper, and (5) concluding or reflecting in the abstract of review articles in linguistics and applied linguistics. Li (2011) also studied the moves of abstracts in chemistry RAs and found five moves, including introduction, purpose, method, product, and conclusion. The “introduction” move involves locating the research by presenting current knowledge and discussing previous research. The “purpose” is aimed to foreground the purpose of the study. “Method” deals with describing the method and presenting information about the subjects, procedures, materials, instruments, and/or the design of the study. “Product” involves briefly summarizing the main findings of the study. “Conclusion” includes final claims about the importance of the study and implications drawn from the findings. Zare and Naseri, 2021a found frequent self-references (*I*, *we*), different levels of both personal and impersonal language styles, and dominant present simple and present perfect tense use as some of the linguistic features of abstracts in review articles.

The introduction as another main section in RAs is used “to justify the study being reported” (Samraj, 2008, p. 56). Swales (1990) showed that RA introductions follow what he termed the CARS model. According to this model, RA introductions feature three important moves: (1) establishing a territory, (2) establishing a niche, and (3) occupying a niche. On the other hand, Tseng (2018) found three moves for theoretical framework sections in language and linguistics papers, i.e. (1) providing a theoretical background, (2) establishing a theoretical framework, and (3) sharpening the significance and/or focus of one’s study that uses the framework. Additionally, Kanoksilapatham (2005) found a set of three moves in the introductions of biochemistry RAs, including (1) announcing the importance of the field, (2) preparing for the present study, and (3) introducing the present study. The first move “announcing the importance of the field” involves (a) claiming the centrality of the topic, (b) making topic generalizations, and (c) reviewing previous research. The second move “preparing for the present study” deals with (a) indicating a gap and (b) raising a question. The third move “introducing the present study” involves (a) stating purpose(s), (b) describing procedures, and (c) presenting findings. The similarity of introductions and theoretical frameworks is mainly because such sections are integrated with one another in most papers. In introductions, the research study is situated within previous studies. Therefore, introductions contain a high number of citations (Swales and Feak, 2012). Additionally, as the findings of previous studies are considered established knowledge and established knowledge should be respected through the use of present tense (Day, 1998), present tense is dominant in this section (Swales and Feak, 2012).

The two other sections in RAs, the results and the discussion sections, which are sometimes integrated, deal with facts and reporting findings, and interpreting and commenting on findings, respectively (Swales and Feak, 2004). Yang and Allison (2003) found six moves for the results section of RAs in applied linguistics, including preparatory information, reporting results, commenting on results, summarizing results, evaluating the study, and deductions from the research, and seven moves for the discussion section, i.e., background information, reporting results, summarizing results, commenting on results, summarizing the study, evaluating the study, and deductions from the research. Peacock (2002) also found eight moves, i.e., information move, finding, expected or unexpected outcome, reference to previous research, explanation, claim, limitation, and recommendation, in the discussion section of RAs in physics and material science, biology, environmental science, business (marketing and management), language and linguistics, public and social administration, and law. Kanoksilapatham (2005) found four moves for the results section of biochemistry RAs. These include (1) stating procedures, (2) justifying procedures or methodology, (3) stating results, (4) stating comments on the results. The first move “stating procedures” involves (a) describing aims and purposes, (b) stating research questions, (c) making hypotheses, and (d) listing procedures or methodological techniques. The second move “justifying procedures or methodology” deals with (a) citing established knowledge of the procedure and (b) referring to previous research. The third move “stating results” involves (a) substantiating results and (b) invalidating results. The last move “stating comments on the results” involves (a) explaining the results, (b) making generalizations or interpretations of the results, (c) evaluating the current findings, (d) stating limitations, and (e) summarizing.

Kanoksilapatham (2005) also found four moves for the discussion section of biochemistry RAs, including (1) contextualizing the study, (2) consolidating results, (3) stating limitations of the study, and (4) suggesting further research. The first move “contextualizing the study” deals with (a) describing established knowledge and (b) presenting generalizations, claims, deductions, or research gaps. The second move “consolidating results” includes (a) restating methodology (purposes, research questions, hypotheses restated, and procedures), (b) stating selected findings, (c) referring to previous literature, (d) explaining differences in findings, (e) making overt claims or generalizations, and (f) exemplifying. The third move “stating limitations of the study” includes (a) limitations about the findings, (b) limitations about the methodology, and (c) limitations about the claims made. The last move “suggesting further research,” which is an optional move, involves making suggestions about further research.

As most of the results section is dedicated to presenting results (Yang and Allison, 2003), past tense is more frequent than present tense in such sections (Swales and Feak, 2012). On the other hand, as the discussion section mainly deals with interpreting results, comparing them with the literature, accounting for and evaluating them, (Yang and Allison, 2003), present simple and present perfect tenses are much more frequent than past tense in such sections (Swales and Feak, 2012). Furthermore, as interpretation and evaluation of results should be done cautiously and writers of RAs need to avoid making strong claims, hedges are more frequent in the discussion than in other sections (Swales and Feak, 2012).

## Materials and Methods

### Corpus Compilation

A corpus of 4 million words comprising 1,185 chemistry research articles (CRAC) was developed for the study. The papers were all downloaded from Elsevier’s online platform “ScienceDirect.” The RAs are equally distributed across the four main subject areas of analytical chemistry, inorganic chemistry, organic chemistry, and physical/theoretical chemistry. In order to select the articles, first, we randomly picked out 10 journals from each subject area. However, as we had access to only eight analytical chemistry periodicals at the time of the study, all these eight journals were selected. This led to a number of 38 JCR-indexed periodicals. Afterward, eight volumes from each journal were selected, except for analytical chemistry journals, for which we selected 10 volumes from each so that we could have the same number of volumes in each area. This led to 320 volumes, published between 2003 and 2009. Later, one issue from each of the 320 volumes was randomly selected, and all the articles, with integrated results and discussion sections, published within these issues, were included in the corpus. The articles were all published under the categories, such as “original article” or “original research.” This led to the selection of more than 1,185 RAs. Next, the texts from each section, i.e., abstract, introduction, results and discussion, were extracted and used to develop three different sub-corpora, i.e., one from abstracts, one from introductions, and one from the results and discussion section. The length of the abstract sub-corpus was 276,136; the introduction sub-corpus was 883,917; and the results and discussion sub-corpus was 2,839,953 words. We excluded the method section from our analysis because it consisted of a large number of formulas and values. Furthermore, interviews with some chemistry students and professors before the study showed that the method section was the least challenging for them.

### Analysis and Classification

A corpus-based discourse analytic approach was followed in this study. A corpus-based approach was followed to generate lexical bundles from the different sections of RAs; a discourse analytic approach was followed to investigate their functions. The corpus-based approach is in line with the first step in Hyland’s (2008b) approach, which involves the identification of lexical bundles, and the discourse analytic approach is in accordance with the second step in his approach, which concerns qualitative functional analyses using concordancers.

Identification of the bundles was based on a frequency cut-off of 20 times per million words and a range or breadth of five papers. Frequency refers to the number of times a certain bundle has to occur in the entire corpus or a sub-corpus to be deemed as a lexical bundle. Range, on the other hand, refers to the number of texts where a particular bundle has to occur in order for it to be counted as a formulaic sequence uncharacteristic of a certain writer. Here, for example, a lexical bundle had to occur in at least five different introductions to be counted as a bundle associated with the introduction section. The range was computed “to guard against idiosyncratic uses by individual speakers or authors” (Biber et al., 2004, p. 376). Biber et al. (2004) and Cortes (2013) also set the range at five. Therefore, a list of four-word and longer sequences occurring at least 20 times per million words in at least five different papers for each section, namely, abstract, introduction, and results and discussion, was created, using WordSmith Tools (Scott, 2009). According to Hyland (2008a), four-word bundles are far more common than five-word strings and offer a clearer range of structures and functions than three-word bundles. In addition, as Cortes (2004) states, many four-word strings “hold 3-word bundles in their structure (as in *as a result of*, which contains *as a result*)” (p. 401). Yet, longer sequences up to 7-word bundles were also identified to see if the four-word bundles overlap. This helped in identifying the difference in types of bundles in different sections. Next, key bundles were identified. In the literature, the key bundles are defined as bundles that are significantly more frequent in one corpus (usually the smaller one), compared with another corpus [usually, the larger one, Reference Corpus (RC)] (Hunston, 2002; Baker, 2006). In this study, the key bundles were those bundles that were significantly more frequent in one section, compared with other sections as RC. WordSmith Tools was used to identify key lexical bundles. The keyness of bundles was computed at a *p* value of 0.000001.

Functional investigation of the lexical bundles was done based on Hyland’s (2008a) taxonomy, namely research oriented, text oriented, and participant oriented. This was mainly because Hyland chose disciplines “to represent a cross-section of academic practice: electrical engineering (EE) and microbiology (Bio) from the applied and pure sciences, and business studies (BS) and applied linguistics (AL) from the social sciences” (p. 361). In his categorization, research-oriented bundles help writers structure their activities and experiences and are used for indicating location (e.g., *in the present study*), procedure (e.g., *the purpose of the*), quantification (e.g., *a wide range of*), and description (e.g., *the surface of the*). Text-oriented bundles deal with the organization of the text and include transition signals (e.g., *on the other hand*), resultative signals (e.g., *it was found that*), structuring signals (e.g., *in the next section*), and framing signals (e.g., *with respect to the*). Participant-oriented bundles deal with the reader or writer of the text and contain stance (e.g., *it is possible that*) or engagement features (e.g., *it should be noted that*). Functional investigation of the bundles was done using a discourse analytic approach where the function of each bundle was determined by investigating its co-text in WordSmith Tools. As Hyland (2008b) notes, “while a corpus can tell us which clusters are frequent, an explanation of why they are frequent can only come from texts” (p. 47). In some cases, a single bundle had multiple functions. Such bundles were classified according to their most common use. Because of the specificity of CRAC, some bundles with new functions, not present in Hyland’s taxonomy, were also found in the corpus. Accordingly, new subcategories were developed to accommodate these new functions.

To avoid the subjectivity inevitable in the qualitative analysis of corpora, all the lexical bundles were investigated independently by each author. In order to test inter-rater agreement, Cohen’s kappa coefficient was calculated. Here, a Cohen’s kappa of 0.79 was computed. Attempts were made to reach full agreement between the authors about the functions of bundles. A third researcher was invited in case disagreements ensued when determining the functions of lexical bundles. For reliability measures, a random sample of the lexical bundles was also functionally investigated by the third researcher, which resulted in a Cohen’s kappa of 0.81.

Finally, the concordance lines, produced for each bundle by WordSmith Tools, were further investigated to find out about their structural features.

## Results

### Key Lexical Bundles in Abstract, Introduction, and Results and Discussion Sections

Functional analysis of lexical bundles in CRAC led to the emergence of two functions for chemistry RAs, which were not present in Hyland’s, (2008a,b) taxonomy. These two functions were added as new subcategories to the research-oriented category in Hyland’s taxonomy. These include purpose-oriented bundles, which specifically refer to the aim/aims of the study and literature-oriented bundles, which are used to refer to the related literature. Narrowing down some of the categories in Hyland’s taxonomy would be useful as the corpus used in the present study is much more specific, compared to the one used in Hyland’s. Altogether, 197 key bundles were identified in the three sections, 1,599 and 83 in abstract, introduction, and results and discussion, respectively. This is in keeping with Biber and Barbieri’s (2007) observation that all university registers consist of lexical bundles but to different extents. In this part, functional categories in each section are described in more detail. To avoid overestimating the number of bundles and to better understand the differences between the three sections, not only did we identify the number of key bundles WordSmith produced for each part, but, also, we determined different types of key bundles in each section. That is, after analyzing each bundle in the concordancer, we discounted those that overlapped and were part of longer strings (e.g., “*is one of the*” and “*one of the most*” were considered as one type because, in most of the cases, they were part of “*is one of the most*”). Furthermore, we considered as one type those bundles that were only different in terms of grammatical words (e.g., *was/were found to be)*.

#### Key Lexical Bundles in the Abstract

Table 1 presents the functional analysis of lexical bundles in the abstract section of RAs.

**TABLE 1:** Functional categories in the abstract section of RAs.

Categories	Sub-categories	No. of bundles	Percentage	No. of different bundles
Research-oriented	Procedure	8	53.33	5
	Location	1	6.67	1
Text-oriented	Resultative	1	6.67	1
Participant-oriented	Stance	5	33.33	3
Total		15	100.0	10

As Table 1 shows, a total of 15 key bundles were identified in the abstract of RAs. This is related to the use of procedure, stance, location, and resultative bundles. Approximately, 60% of all the key bundles in this section were research oriented. Among them, most such bundles mainly dealt with a procedure.

Procedure bundles are research-oriented bundles used for structuring experiences and activities. Eight procedure bundles were found as the key in the abstract, four of them are part of the longer string “*have been synthesized and characterized by elemental*.” Hence, there were five different types (see Supplementary Appendix 1A). The bundle “*have been determined by*” was the most key formulaic expression among procedure bundles. These bundles typically appear in two structures: present perfect passive and past passive (1) (*have/has been/were/was synthesized and characterized*). This group of bundles was not among high frequency words in the whole corpus. In RC, they mostly appeared in the introduction section.

The yttrium and lutetium complexes *have been characterized by* X-ray diffraction analysis.

Location bundles are a subcategory of research-oriented bundles that are used to indicate time and place (2) (Hyland, 2008a,b) (see Supplementary Appendix 1B). There was one key bundle of this type in the abstract, i.e., *for the first time*, mostly used to stress the novelty of the finding(s), the new method(s), or material(s), employed in the study.

*For the first time*, a Doering–LaFlammeallene synthesis method was adopted, and the structure was confirmed by monocrystal X-ray diffraction.

Resultative bundles are text-oriented bundles that are used to establish causative or inferential relations between factors (3). There was only one bundle of this type in the abstract section (see Supplementary Appendix 1C). This bundle is part of a larger bundle “*the results show that the*,” which was also a key bundle in this section. Academic writers in chemistry RAs used this bundle to refer to the outcomes of their studies.

The results show that the minimum outlet NOx emission appears at the maximum Ldav and teav.

Stance bundles fall under the participant-oriented category in Hyland’s, (2008a,b) functional taxonomy. Five four-word bundles and three types were found as the key in this section. These bundles convey the writer’s stance on the following proposition (4). Two of these bundles “*was found to be*” and “*were found to be*” were counted as one type because they are just different grammatically. They convey some degree of logical possibility toward the following argument and show the writer’s tentativeness toward what is reported (Cortes, 2004). However, the other three stance bundles, i.e., “*it was found that*,” “*was found that the*,” and “*it is shown that*” seem to encode the following arguments as a fact. The first two bundles are part of “*it was found that the*,” which was also a key bundle in the abstract. Except for “*it is shown that*,” all the bundles in this group were quite frequent in the whole corpus. Writers have used impersonal language, i.e., passive structure, in all these bundles to express their arguments and claims (see Supplementary Appendix 1D).

The selectivity coefficients for different cations determined by the mixed solution method were found to be less than unity.

#### Key Lexical Bundles in the Introduction

Table 2 presents the results of the functional analysis of lexical bundles in the introduction section.

**TABLE 2:** Functional categories in introduction.

Categories	Sub-categories	No. of bundles	Percentage	No. of different bundles
Research-oriented	Quantification	11	10.90	9
	Purpose-oriented	5	4.95	4
	Description	3	2.97	3
	Literature-oriented	21	20.79	11
	Location	2	1.98	1
	Procedure	14	13.86	10
Text-oriented	Structuring signals	29	28.71	20
	Framing signals	7	6.94	7
Participant-oriented	Stance features	7	6.93	3
Total		99	100.0	68

Totally, 99 key bundles were identified in the introduction section of chemistry RAs, as Table 2 shows. Among the three main functional categories, over 55% of the bundles were research oriented. Structuring signals, followed by literature-oriented bundles, among the subcategories, were the most frequently used bundles in the introduction. A detailed discussion of the functions of bundles in the introduction section of RAs follows.

Quantification bundles are a subcategory of research-oriented bundles in Hyland’s, (2008a,b) functional taxonomy. These bundles give information about the number, amount, variety, or degree of the elements following them (5). Eleven four-word bundles with 9 different types were found in this section.

Fabrication of self-assembled monolayer (SAM) coatings is one of the most successful approaches to chemical modification.

Among quantification bundles, “*one of the most*” and “*is one of the*,” which are part of the longer sequence “*is one of the most*” took the leading positions. In most of their occurrences, they follow words like “*is*,” “*as*,” and “*was shown to be*,” and they are followed by adjectives, such as “*important*,” “*powerful*,” “*widely used*,” “*useful*,” “*interesting*,” “*common*,” and “*effective*.” These two bundles were among the first 100 most frequent bundles in the whole corpus, too. It was also found that the bundle “*of the most important*” is mostly part of the string “*is one of the most important*,” so the three bundles were considered as one type. The other two bundles “*a wide range of*” and “*a wide variety of*” usually follow the words “*over*” and “*in*.” Structurally, all the quantifying bundles, except for “*is one of the*,” had the structures of “prepositional + of” and “noun phrase + of” (see Supplementary Appendix 2A). In RC, the bundles in this group were more frequent in the results and discussion section compared to the abstract.

Some lexical bundles in the introduction section of RAs have been specifically used to refer to the aim/aims of the study. A new subcategory, called purpose-oriented bundles, was created as a subcategory of the research-oriented category in Hyland’s, (2008a,b) functional taxonomy (6) to embrace these bundles. Totally, five four-word bundles in four different types were found as a key in the introduction section (see Supplementary Appendix 2B). These bundles are commonly used at the beginning of the introduction. That is, writers in chemistry RAs state the purpose/purposes of their studies at the beginning of this section. All these bundles have words like *purpose*, *aim*, or *objective* in their structure and appear in larger five-, six- and seven-word bundles, such as “*the aim of this study/work is/was to, the aim of the present study/paper/work is/was to, the purpose of this study/paper/work is was to*, and *the objective of this study*/*paper/work is/was to*.” An eight-word bundle was also identified as a key in this section, i.e., “*the aim of the present work is to*.” Structurally, all purpose-oriented bundles followed the pattern of “noun phrase + of,” usually followed by “*is/was to*.” Gledhill (2000a) also refers to such expressions as “purpose oriented.”

The purpose of this work is to determine whether the presence of the angularly fused pyran ring is present in acronycine.

Description bundles are used to describe some characteristics of the elements following them, such as form, function, size, content, etc. (7). Three types of bundles were used in the introduction section for description (see Supplementary Appendix 2C). Among these, “*the coordination chemistry of*” was the most key bundle in this group, which seems to be specific to the discipline. Structurally, all description bundles followed the pattern of “noun phrase + of.”

Many lignans that are considered interesting lead structures for the development of new anti-tumoral drugs.

A group of 20 four-word lexical bundles in 11 different types was found in the introduction section, which was used to refer to the related literature (8) (see Supplementary Appendix 2D). These bundles were not present in Hyland’s, (2008a,b) functional taxonomy and are listed as a new subcategory of research-oriented bundles, i.e., literature oriented. Gledhill (2000b) refers to them as biochemical reports. Many of these bundles occurred only in the introduction section, as they appeared very infrequently, one time or two times, in the RC. Many of these bundles have passive structures. This is related to the use of impersonal language in reporting the literature when the raised issue is not a subject of disagreement. Additionally, except for one bundle, all such bundles are in present perfect (Swales and Feak, 2012).

The essential oil from the fruits and fruit pericarp *has been the subject* of study by several investigators.

Analysis of the concordance lines of these bundles showed that many of these bundles are part of longer five- to seven-word strings. Examples are “*much attention has been paid to the*,” “*have been devoted to the*,” “*have been the subject of (many)*,” “*have received considerable attention in recent years*.” Moreover, writers of chemistry RAs used words like “*widely*” and “*extensively*” before the word “*studied*” and other words, such as “*considerable/much*” before “*attention*” to stress the importance of the topic under investigation and the attention it has received recently from the researchers.

Regarding location bundles, there were only two four-word bundles in the introduction section, and both referred to particular duration of time (see Supplementary Appendix 2E). These bundles only appeared in this section and did not occur in the reference corpus at all. They are part of the five-word bundle “*in the last few years*,” which was also found as a key in this section. It is worth noting that this bundle is mostly seen in contexts in which authors state that the given topic has recently been the focus of many researchers (9). In fact, it mainly appears in the same contexts as literature-oriented bundles.

Controlled drug delivery products, using biocompatible or biodegradable polymers, have received considerable attention in the last few years.

Eleven procedure bundles (10 different types) were found as a key in the introduction section (see Supplementary Appendix 2F). These bundles have been used in the introduction section of chemistry RAs to describe the instruments, methods, manners, or materials used in research (10). The use of passive form is a shared feature of most of these bundles. Additionally, except for one bundle, i.e., *have been developed for*, all these bundles have the word “*use*” as one of their constituents. Most of these bundles are structurally passive and are followed by prepositions like “*in*,” “*for*,” “*as*,” and “*to*.” Also, the word “*widely*” is one constituent of most of these bundles. In RC, these bundles appeared more in the results and discussion section than in the abstract.

In addition to the determination of urea, urease-based biosensors have been widely used for the determination of heavy metals in environmental and biological samples.

Twenty-nine four-word structuring bundles in 20 different types were found as a key in the introduction section (see Supplementary Appendix 2G). Structuring bundles are “text-reflexive markers, which organize stretches of discourse or direct reader elsewhere in text” (Hyland, 2008a, p. 14). In other words, these bundles give notice of text stages, mark the organization of texts, and direct readers to the particular parts of the texts so that they can find the information they need for a better understanding of the content (11) (Hyland, 2008a,b).

In the present paper, we report for the first time the separation of PE–PP blends by high-temperature gradient HPLC.

The bundle “*in this paper we*” was the most key bundle in this section. The first part of these bundles consists of words like “*in this*,” “*in the present*,” “*herein*,” and “*of this/the present*,” all of which point to the paper under study, followed by words such as “*paper*,” “*work*,” “*study*,” and “*article*,” suggesting that writers refer to their own research. The bundles that start with “*in this*,” “*in the present*,” and “*herein*” are followed by the word “*we*.” Some of these bundles expand to longer sequences, such as “*in this paper/work we report the*,” “*in the present paper/work we*.” Those bundles that begin with “*of this/the present*” are followed by “*is/was to*.” Analysis of the concordance lines of these bundles showed that they follow purpose-oriented words like “*the aim/purpose/goal*” and expand to a longer sequence “*the aim/purpose/goal of this/the present study/paper/work/article is/was to*.” Structuring bundles are followed by words, such as “*describe*,” “*report*,” “*investigate*,” “present,” “*explore*,” “*focus on*,” and “*expand on*.” In fact, this group is typically preceded or followed by the arguments that express the subject and/or the purpose/purposes of the study, reported by the writer. Many of these bundles were extremely infrequent in the RC. However, those bundles that also appeared in RC were used in both abstract, and results and discussion sections. Some five- and six-word key bundles were also found in this section (see Supplementary Appendix 2G).

Framing bundles are used to “situate arguments by specifying limiting conditions” (Hyland, 2008a, p. 14). These bundles restrict the scope of an argument by relating it to narrower arguments (12).

Carbamazepine is a well-established drug used in the treatment of epilepsy.

There were seven bundles of this type in the introduction section of chemistry RAs (see Supplementary Appendix 2H). More importantly, all these bundles were prepositional phrases with “*of*.” The bundles “*for/in the treatment of*” are mostly followed by the names of diseases, such as *asthma, Alzheimer, cancer*. In RC, these bundles were mostly seen in the results and discussion section, compared to the abstract.

Stance bundles in the introduction section included six four-word key bundles (see Supplementary Appendix 2I). The first four bundles in this group convey the writer’s knowledge of the lack of any previous study on the subject under investigation (13). They expand to the 6-word bundle “*to the best of our knowledge*.” These bundles were usually used at the end of the introduction section to reflect the novelty and significance of the study by indicating that it focuses on a new aspect of the subject area, which has not been explored by other researchers yet. In RC, they appeared in the results and discussion much more frequently than in the abstract. “*There is a need*” is another example of this group, which is an emphatic stance bundle stressing the need for more studies on the subject (14).

To the best of our knowledge, polyesters end-capped with trimethoxysilyl groups have not been described before.
Clearly, there is a need for additional studies of biomass and nutrients in alpine tundra.

#### Key Lexical Bundles in the Results and Discussion Section

Overall, 83 formulaic expressions were found as key lexical bundles in the results and discussion section of chemistry RAs. Table 3 presents the functional categorization of these bundles.

**TABLE 3:** Functional categories in results and discussion.

Categories	Sub-categories	No. of bundles	Percentage	No. of different bundles
Research-oriented	Descriptive bundles	8	9.30	8
Text-oriented	Structuring signals	18	20.93	16
	Resultative signal	8	9.30	6
	Framing bundles	11	12.79	11
	Transition signals	1	1.16	1
Participant-oriented	Stance features	29	33.72	19
	Engagement features	11	12.79	7
	Total	86	100	68

As Table 3 shows, stance bundles were the most frequent bundles, followed by structuring signals. Eight key four-word description bundles were found in the results and discussion section (see Supplementary Appendix 3A). Some of these bundles seem to be specifically related to the field of chemistry. These include “*the dihedral angle between*,” “*the IR spectra of*,” and “*the spectra of the*.” Most of these bundles follow the structural pattern of “noun phrase + of” (15). In RC, they occurred more in the introduction than in the abstract.

The general features of the IR spectra of complexes 1–3 are similar in nature.

Regarding structuring lexical bundles, 18 four-word bundles of this type were identified as a key in the results and discussion section (see Supplementary Appendix 3B). The words “*table*” and “*figure*” are used as one of the main constituents of most of these bundles. Writers have used these bundles to direct readers to tables and figures in order to help them better understand what has just been reported (16).

All of the imaginary frequencies of the transition states are listed in Table 2.

The bundle “*can be seen in*” appeared 156 times in the results and discussion section and only 10 times in the reference corpus. This bundle expands into the six-word bundle “*as can be seen in table/figure*.” Likewise, the bundles “*can be seen from*” and “*results are shown in*” are almost always followed by the words “*figure*” and “*table*” in this section. Structurally, most of these bundles had the structure of “passive verb + prepositional phrase,” and almost all their occurrences in the RC were in the introduction, rather than the abstract. This group of bundles is preceded by words like “*as*” and “*are/is*.” Bundles that begin with *are/is* were more frequent in the RC than those which start with “*as*,” and they have more diverse verbs in their structure (e.g., *shown, presented, listed, summarized*, and *reported*), compared to those that follow “*as*,” which contain two words “*shown*” and “*seen*” as their main verbs. All the cases of these bundles follow a passive voice structure.

Eight key four-word resultative bundles in 6 types were identified in the results and discussion section of RAs (see Supplementary Appendix 3C). The bundle “*as a function of*,” which was the most key bundle in this group, was also the third most frequent bundle in the whole corpus (with more than 800 occurrences). Among these, “*effect of ph on*,” which establishes causal relationships between factors, parts, or arguments, was the least frequent in RC. The word “*due*,” which appeared about 2,900 times in the result and discussion section is always followed by the preposition “*to*.” In this section, the bundles “*is due to the*” (17), “*to the presence of*” (18), and “*due to the presence*” (19) are parts of the six-word bundle “*is due to the presence of*,” which was also identified as a key. In RC, these bundles appeared in both sections but slightly more frequently in the introduction.

The increased of NLC size by increasing the drug charge *can be due to the* higher viscosity of the molten oil phase because the mp of the drug is higher than the mixing temperature.
The peak for carbon was observed *due to the presence of* carbon coating on the sample holder.
It is known that the excellent solubility associated with these polyimides *might be due to the presence of* the introduction of the cycloaliphatic unit into the polyimide backbone would facilitate less polymer–polymer interaction (18).

Regarding framing signals, 12 different key bundles were found in the results and discussion section of chemistry RAs (see Supplementary Appendix 3D). All the bundles of this type were structurally prepositional phrases (20). In RC, these bundles were more frequent in the introduction than in the abstract. In this group, some of the bundles were among the first 20 high-frequency bundles in the whole corpus. For example, “*in the presence of*,” with more than 1,300 occurrences, was the first most frequent bundle in the whole corpus, followed by “*in the case of*,” with 1,100 occurrences. Also, “*With respect to the*” and “*to the formation of*” were among the first 20 frequent bundles in the corpus as a whole.

Further investigations were carried out in the presence of various model olefins, in particular 1-pentene, 1-octene, and 1-dodecene.

Regarding transition signals, there was only one such bundle in the results and discussion section of RAs, i.e., “*on the other hand*” (see Supplementary Appendix 3E). According to Hyland (2008a; 2008b), transition bundles “establish additive or contrastive links between elements.” This lexical bundle has been used to connect two propositions and clarify that the latter proposition is in contrast with the former (21). In the whole corpus, it took the fourth position among frequent bundles. In RC, it was much more frequent in the introduction than in the abstract.

The stabilizer possesses more CO2-philic groups to assure solubility in the continuous phase and thus provides less stability to the latex with weaker anchoring onto the particle surface. *On the other hand*, the copolymer of lower FOMA content was turned out to be too hydrocarbon-philic to act as an effective stabilizer for the dispersion polymerization.

There were 29 key four-word stance bundles with 19 different types in the results and discussion section (see Supplementary Appendix 3F). Such bundles as “*it is clear that*,” “*it is obvious that*,” and “*the fact that the*” reflect the writers’ overt stance and certainty toward the following proposition (22).

It is obvious that the prediction yields satisfactory agreement with a relative error for the copper concentration below 10%.

Other formulaic expressions, such as “*is in good agreement*,” “*is close to the*,” “*this is consistent with*” were also considered as stance bundles, as they were used to compare one part or argument with another, and thus denote the writer’s attitudes and evaluations toward the text (23).

According to these results, chitosans derived from lobster chitin are similar to those of the commercial samples evaluated.

There were other stance bundles in this category that help writers express their argument/arguments as opinions rather than facts (24). Using these bundles, writers express logical possibilities for the following propositions. In fact, they take a more tentative stance toward what is being reported and allow for the possibility of potential alternative explanations. Structurally, stance bundles were typically clausal. Words such as “*may*,” “*might*,” and “*can*” have been used in these bundles to hedge the effect of an affirmation, or to make an affirmation or argument more tentative.

The peak at 820 C *can be assigned to* decomposition of calcium carbonate.

There were some bundles in this group, which refer to the consistency of the obtained results with previous studies. These include “*is consistent with the*,” “*are similar to those*,” “*is close to the*,” and “*in agreement with the*.” Among these bundles, those which contain the word “*agreement*” are parts of longer strings “*is in agreement with the*” and “*is in good agreement with the*.” These two bundles usually follow “*the result*” and, sometimes, “*which*” and “*this.*” Analysis of the concordance lines of “*is in good agreement with the*” showed that authors also use other words such as “*excellent*,” “*close*,” and “*f’ull*” instead of “*good*” to show the high consistency of their results with those of the previous studies. It is worth noting that, in this group, bundles that show the writer’s attitude toward the cause(s) of the obtained result are usually followed by the preposition “*to*” (e.g., “*it is/can/may/might be assigned/attributed/due/related to the*”).

Eleven key four-word bundles with seven types were found for engagement features in the results and discussion section (see Supplementary Appendix 3G). “*It can be seen*” was the most frequent bundle in this section. Many of engagement bundles are part of larger five- and word-word bundles. For example, “*it can be seen*” is extended to “*it can be seen that*,” and “*it can be seen that the*.” Other longer sequences like “*as can be seen from*,” “*it should be noted that*,” and “*it can be observed that*,” which incorporate many of the bundles in this group were also found as a key in this section. Most of the bundles of this type are preceded by “*it*” and followed by “*that*.” Analysis of the concordance lines of these bundles showed that bundles that begin with “*as*” are usually followed by “*in*” or “*from*” and then “*table/figure*” (*as seen/can be seen in figure/table, as seen/can be seen from figure/table*). The words “*seen*,” “*can*,” and “*observed*” are the main constituents of this group of bundles. Engagement bundles have been used mostly to engage readers as participants in tasks or observers of particular parts of the text. Writers have used bundles of this type for indicating the importance of points and necessitating the readers’ attention to these (25), or guiding the readers to particular parts of the text or argument (26).

It should be noted that, under practical operating conditions, vegetable oil will not be miscible with SCCO2.
It can be observed from Table 1 that absorption bands exhibit very little positive solvatochromism.

### Comparison of the Three Sections in Terms of Number and Type of Bundles

Table 4 presents the number of bundles WordSmith produced and the number of different types of bundles in each section.

**TABLE 4:** Comparison of the abstract, introduction, and results and discussion sections in terms of number and type of bundles.

	No. of bundles	Types of bundles	% for bundle types	Total frequency
Abstract	15	10	0.0036	312
Introduction	99	68	0.0076	3019
Results and discussion	86	68	0.0023	1,1538

Considering the size of each section (abstract: 276,136 words; introduction: 883,917 words; results and discussion: 2,839,953 words), although results and discussion relied on a larger number of bundles, compared to other sections, the introduction incorporated more different bundle types than the two other sections, as Table 4 shows.

### Functional Comparison of Bundles in the Abstract, Introduction, and Results and Discussion

Table 5 shows the results of the comparison of abstract, introduction, and results and discussion sections in chemistry RAs in terms of the use of three major functional categories.

**TABLE 5:** Comparison of sections in terms of the major functional categories.

Section	Research-oriented (%)	Text-oriented (%)	Participant-oriented (%)
Abstract	60.00	6.67	33.33
Introduction	55.45	35.65	6.93
Results and discussion	9.30	44.18	46.51
			

As can be seen in Table 5, most lexical bundles in the abstract and introduction sections were research oriented. For results and discussion, however, most bundles were either participant oriented or text oriented. Text-oriented bundles were the least frequently used formulaic expressions in the abstract; participant-oriented bundles were the least frequently used bundles in the introduction; and research-oriented bundles were the least frequent expressions in the results and discussion section of chemistry RAs.

In order to investigate further differences between the three sections, the three sections were compared in terms of functional subcategories.

#### Comparison of the Three Sections in Terms of Research-Oriented Subcategories

As mentioned earlier, research-oriented bundles constituted the majority of key bundles in the introduction. However, they were the least used bundles in the results and discussion section. Table 6 presents research-oriented bundle subcategories in the three RA sections.

**TABLE 6:** Research-oriented subcategories in the three sections.

Research-oriented functions	Abstract	Introduction	Results and discussion
Location	1 (6.67%)	2 (1.98%)	–
Procedure	8 (53.33%)	14 (13.86%)	–
Quantification	–	11 (10.90%)	–
Description	–	3 (2.97%)	8 (9.30%)
Purpose markers	–	5 (4.95%)	–
Literature-oriented	–	21 (20.79%)	–

As Table 6 shows, research-oriented bundles were limited to description in the results and discussion section; in the abstract, these bundles were related to either location or procedure. While procedure bundles were used very frequently in the abstract and introduction sections, no bundle of this type was found in the results and discussion section. That is, chemistry RA writers described the materials, methods, and techniques of their own research in the abstract and those of prior research in the introduction section.

Furthermore, literature-oriented bundles comprised more than 21% of all the key bundles in the introduction section. Although completely absent in the abstract and results and discussion sections, it cannot be concluded that the related literature is reviewed and reported only in the introduction section of chemistry RAs, as some connections are drawn to prior research in the results and discussion sections.

#### Comparison of the Three Sections in Terms of Text-Oriented Subcategories

Table 7 shows text-oriented bundle subcategories in the three sections of chemistry RAs.

**TABLE 7:** Text-oriented subcategories in the three sections.

Text-oriented category	Abstract	Introduction	Results and discussion
Transition signals	–	–	1 (1.16%)
Resultative bundles	1 (6.67%)	–	8 (9.30%)
Structuring signals	–	29 (28.71%)	18 (20.93%)
Framing signals	–	7 (6.94%)	11 (12.79%)
			

As Table 7 shows, text-oriented lexical bundles in the abstract were mainly resultative, i.e., “*the results show that*.” The introduction section, however, lacked key bundles of this type. Additionally, whereas there were no structuring bundles in the abstract, these bundles accounted for most of the text-oriented bundles in both the introduction and results and discussion sections. Apart from the shared reliance on structuring bundles more than other subcategories of the text-oriented category, the introduction and results and discussion sections used these bundles in different ways. In the introduction, structuring signals were predominantly used by writers to refer to their own studies. On the other hand, in results and discussion, the writers used these bundles to direct readers to visual parts of their text, i.e., tables and figures. Structurally, most of these bundles were “passive + prepositional phrases” in results and discussion, and “prepositional phrase + other post modifiers” and “pronoun/noun + be” in the introduction. Furthermore, there were no key framing bundles in the abstract. These bundles, which are used to narrow down the following argument, however, constituted the second most frequent text-oriented bundles in both results and discussion and introduction sections.

#### Comparison of the Three Sections in Terms of Participant-Oriented Subcategories

Table 8 shows participant-oriented bundle subcategories in the three sections of chemistry RAs.

**TABLE 8:** Participant-oriented subcategories in the three sections.

Participant-oriented category	Abstract	Introduction	Results and discussion
Stance features	5 (33.33%)	7 (6.93%)	29 (33.72%)
Engagement features	–	–	11 (12.79%)

As Table 8 shows, the abstract and introduction sections of RAs totally lacked key engagement bundles. These bundles, however, were used frequently in the results and discussion section. As regards to stance bundles, these bundles were used in all the three sections of RAs. The use of these bundles was somehow similar to their use in the abstract and results and discussion. In the abstract, all the stance bundles encoded an impersonal meaning in that they expressed an argument without explicitly identifying the writer of that argument (e.g., *it is shown that*). Two stance bundles in this section reflected the tentativeness of the writer toward the following proposition (e.g., “*was/were found to be*”*).* Three out of five of key stance bundles in the abstract encoded the following argument as a fact (e.g., *it was found that*, *the results show that*). In the results and discussion, on the other hand, more than 42% of all the stance bundles reflected the uncertainty of the writer toward the following proposition. These bundles contained some hedging words such as “*can*,” “*may*,” and “*might*” (e.g., *may be attributed to*). No stance bundle in the abstract contained such hedging words. In addition, more than 39% of stance bundles in the results and discussion section reflected the writer’s attitudes and evaluations of the following proposition (e.g., *are similar to those, it is interesting to)*. Four out of 28 stance bundles in this section clearly indicated the certainty of the writer toward the following argument/arguments (e.g., *it is obvious that)*.

As Table 8 shows, key bundles, which had stance functions in the results and discussion section, were much more frequent than bundles with engagement functions.

## Discussion and Conclusion

The present study was an attempt to investigate lexical bundles in different sections in chemistry RAs, i.e., abstract, introduction, and results and discussion. With its corpus-based nature and narrow-angle analysis, any generalized conclusion is necessarily tentative. Nevertheless, the study resulted in some observations that are worth noting.

First and foremost, the results showed that different sections of chemistry RAs make use of diverse sets of lexical bundles. This is in keeping with the findings of Gledhill (1995, 1996) for cancer RAs. This finding may be attributed to the fact that different sections in RAs deal with different communicative functions (Swales, 1990; Hyland, 2000; Peacock, 2002; Yang and Allison, 2003; Tseng, 2018). Second, two research-oriented functions, i.e., purpose-oriented bundles, which specifically refer to the aims of the study, and literature-oriented bundles, which are used to refer to the related literature, emerged in the functional analysis of introductions for chemistry RAs. The exclusive presence of these bundles in the introduction section supports Swales’ (1990) CARS model and the fact that the nature of this section in RAs is to situate the research within previous studies and introduce its purposes (Swales, 1990). Third, in accordance with the rhetorical functions of abstracts, introductions, and results and discussion sections (Swales, 1990; Hyland, 2000; Peacock, 2002; Yang and Allison, 2003; Samraj, 2008; Zare and Naseri, 2021a), most bundles in the abstract and introduction sections were research oriented due to the dominant presence of procedure bundles in the abstract and literature-oriented bundles in the introduction, whereas, in the results and discussion section, most bundles were either participant oriented or text oriented due to the dominance of structuring signals and stance features in such sections, respectively. This may be attributed to the fact that the results and discussion section mostly deals with presenting the findings and commenting on them (Yang and Allison, 2003). Fourth, stance bundles outnumbered resultative bundles in chemistry RA abstracts. As both were used to show the outcome of research in the abstract, yet stance bundles were all passive, whereas resultative bundles were active, it stands to reason that chemistry RA writers draw more on impersonal language style than personal in the abstract. Fifth, one key location bundle, i.e., *for the first time*, was found exclusively in the abstract section. This may be construed as an attempt to promote and sell the research. As Yakhontova (1997) highlights, writers have to “sell” rather than “tell” their research (as cited in Van Bonn and Swales, 2007). This is even more serious in chemistry because of the large number of research papers published every year. As abstracts are freely accessible, the use of this bundle by chemistry RA writers can be seen as an attempt to publish and promote their research. Sixth, words such as “may,” “might,” and “can” were used in stance bundles in the results and discussion section. This is consistent with the fact that hedges are more frequent in the discussion than in other sections (Swales and Feak, 2012). Seventh, resultative bundles, which signaled association between elements or arguments, were found as a key in the abstract and results and discussion, but not in the introduction section. This reflects the descriptive nature of the language of results and discussion in RAs (Swales and Feak, 2004). Eighth lexical bundles with engagement functions were only found in the results and discussion section. This may indicate that engaging the reader in the text in the results and discussion section where important findings, points, and facts are presented (Swales and Feak, 2004) is much more important than in the abstract and introduction sections. This may also lend support to the set of moves Kanoksilapatham (2005) found for the results, including stating procedures, justifying procedures or methodology, stating results, and stating comments on the results, and discussion sections, i.e., contextualizing the study, consolidating results, stating limitations of the study, and suggesting further research, in biochemistry RAs. Ninth, structuring bundles were used both in the introduction and results and discussion but in different ways. Given the function of structuring bundles, i.e., to mark the organization of the text, they may be present in different sections in RAs with different forms. For example, in the results section, they mostly refer to tables and figures, whereas, in the introduction, they are used in statements that refer to the purpose of the study. Finally, in the results and discussion section, the bundles dealing with stance were much more frequent than the bundles with engagement functions. This may indicate that writers in chemistry tend more to express their attitudes and stance toward the proposition than to engage the reader in the text, in the results and discussion section of their RAs.

Altogether, these findings mainly suggest that various sections in chemistry RAs are associated with specific sets of lexical bundles and, as such, deal with different rhetorical functions (Tseng, 2018). This resonates Kanoksilapatham’s (2005) observation of moves in biochemistry RAs. Kanoksilapatham found different sets of moves for different sections in RAs.

Structurally, the findings showed that the form and function of many of these bundles are closely related. For example, bundles that were used as stance bundles in the abstract and results and discussion sections were mostly clausal, with “passive verb + prepositional phrase” and “be + noun/adjective” structures. This is in keeping with Conrad and Biber’s (2005) observation that common four-word bundles expressing stance in academic prose are all impersonal. Additionally, most of the structuring signals in results and discussion had the “passive + prepositional phrase” structure. Such bundles were mostly of “prepositional phrase + other post modifiers” and “pronoun/noun + be” type, with the plural first-person pronoun “*we*” in the introduction. Moreover, all the descriptive bundles in results and discussion had the structure of “noun phrase + of.” Furthermore, all the quantifying bundles in the introduction, except for “*is one of the*,” followed “noun phrase + of” and “prepositional phrase + of.” In this section, also, literature-oriented bundles mostly had the structure “passive + prepositional phrase”; all the purpose-oriented bundles were of “noun phrase + of” type; and all the framing signals followed the “prepositional phrase + of” structure. These findings mirror Hyland’s (2008a) observation that research-oriented bundles mainly feature noun phrases and text-oriented bundles primarily consist of prepositional phrases. Also, these findings are, generally, in line with those of Biber et al. (1999, 2004), Cortes (2002, 2004, 2013), Qin (2014), and Pan et al. (2016), Zare and Naseri (2021b) who found that academic prose primarily relies on phrasal rather than clausal bundles. “Careful integration of information in academic prose requires the use of noun phrases and prepositional phrases, which leads to a shift from clausal style to phrasal style in academic prose,” as Pan et al. (2016, p. 65) note. Overall, these findings mirror the results of previous studies that pointed to the existence of a close association between the form and function of lexical bundles (e.g., Biber et al., 2004; Biber and Barbieri, 2007; Zare and Naseri, 2021b). Such an association between the form and function of bundles suggests that they are stored as wholes in memory and are used as single units (Biber et al., 2004).

Theoretically, the purpose of this research was to discover the language of different sections in chemistry RAs. Hence, the list of bundles identified in this study, along with their lexical and structural features in different sections of chemistry RAs, may be used as a basis for comparative research on other languages, disciplines, and registers. Pedagogically, similar to other EAP studies, conducting this study was motivated by the need to advise EAP learners, teachers, and material developers. Therefore, EAP materials developers may use the derived lexical bundles in preparing materials for academic English reading and writing in chemistry. Additionally, EAP teachers who teach academic English reading and writing to chemistry students need to orient their syllabi to these sequences and raise their students’ awareness of such formulaic sequences and their rhetorical functions. The findings of this study may also find practical application in EAP testing. EAP test developers may also incorporate the bundles into their tests on academic English reading and writing in chemistry. Finally, the results may be incorporated into instructional materials, used for English for Research Publication Purposes courses in chemistry. Future studies need to investigate the use of lexical bundles in different sections of RAs in other languages, disciplines, and registers.

## Data Availability Statement

The raw data supporting the conclusions of this article will be made available by the authors, without undue reservation.

## Author Contributions

Both authors listed have made a substantial, direct, and intellectual contribution to the work, and approved it for publication.

## Conflict of Interest

The authors declare that the research was conducted in the absence of any commercial or financial relationships that could be construed as a potential conflict of interest.

## Publisher’s Note

All claims expressed in this article are solely those of the authors and do not necessarily represent those of their affiliated organizations, or those of the publisher, the editors and the reviewers. Any product that may be evaluated in this article, or claim that may be made by its manufacturer, is not guaranteed or endorsed by the publisher.
