# Supplementary material for: Lexical Bundles in Chemistry Research Articles
Source: Front Psychol. 2022 May 25;13:906641. doi: 10.3389/fpsyg.2022.906641 (PMC9174748; doi:10.3389/fpsyg.2022.906641)
Supplement: Supplementary file 1 [file Data_Sheet_1.docx]

Appendixes

(Please note that in the following tables, lexical bundles longer than four words are also provided.)

Appendix 1.a

Procedure bundles in abstract

| Lexical bundles | Freq. in abstract | Normed occurrence | Freq. in RC | Keyness score |
| --- | --- | --- | --- | --- |
| have been determined by | 13 | 0.006936 | 7 | 56.89061 |
| has been studied by | 15 | 0.008003 | 14 | 55.87035 |
| have been synthesized and | 15 | 0.008003 | 15 | 54.53619 |
| been synthesized and characterized | 12 | 0.006402 | 8 | 49.63288 |
| synthesized and characterized by | 10 | 0.005335 | 5 | 44.55455 |
| have been characterized by | 7 | 0.003732 | 0 | 44.25169 |
| was developed for the | 10 | 0.005321 | 6 | 42.56658 |
| and characterized by elemental | 8 | 0.004265 | 2 | 40.73863 |

Appendix 1.b

Location bundles in abstract

| Lexical bundles | Freq. in abstract | Normed occurrence | Freq. in RC | Keyness score |
| --- | --- | --- | --- | --- |
| for the first time | 24 | 0.0128 | 83 | 45.00048 |

Appendix 1.c

Resultative bundles in abstract

| Lexical bundles | Freq. in abstract | Normed occurrence | Freq. in RC | Keyness score |
| --- | --- | --- | --- | --- |
| The results show that | 19 | 0.0101 | 31 | 56.392 |

Appendix 1.d

Stance bundles in abstract

| Lexical bundles | Freq. in abstract | Normed occurrence | Freq. in RC | Keyness score |
| --- | --- | --- | --- | --- |
| it was found that | 49 | 0.0261 | 201 | 79.778 |
| it is shown that | 19 | 0.0101 | 21 | 66.585 |
| was found to be | 53 | 0.0282 | 321 | 57.636 |
| was found that the | 30 | 0.0160 | 106 | 55.314 |
| it was found that the | 30 | 0.0160 | 106 | 55.314 |
| were found to be | 28 | 0.0149 | 136 | 38.879 |

Appendix 2.a

Quantification bundles in introduction

| Lexical bundles | Freq. in introduction | Normed occurrence | Freq. in RC | Keyness score |
| --- | --- | --- | --- | --- |
| one of the most | 104 | 0.016855 | 47 | 236.84 |
| is one of the | 104 | 0.016823 | 68 | 199.55 |
| a wide range of | 80 | 0.012965 | 78 | 119.78 |
| a wide variety of | 47 | 0.007607 | 16 | 118.69 |
| a large number of | 45 | 0.007284 | 49 | 61.962 |
| one of the most important | 24 | 0.003885 | 14 | 48.798 |
| in a variety of | 24 | 0.003885 | 16 | 45.574 |
| of the most important | 25 | 0.004047 | 19 | 44.081 |
| for a number of | 17 | 0.002752 | 8 | 38.075 |
| an important class of | 11 | 0.001781 | 1 | 36.781 |
| a great number of | 11 | 0.001782 | 1 | 36.781 |
| of a number of | 18 | 0.002913 | 11 | 35.765 |

Appendix 2.b

Purpose-oriented bundles in introduction

| Lexical bundles | Freq. in introduction | Normed occurrence | Freq. in RC | Keyness score |
| --- | --- | --- | --- | --- |
| the aim of this | 40 | 0.006474 | 16 | 95.486 |
| aim of the present | 29 | 0.004694 | 13 | 66.391 |
| the aim of the | 22 | 0.003561 | 6 | 66.254 |
| the aim of the present | 22 | 0.003561 | 6 | 59.434 |
| the purpose of this | 21 | 0.003399 | 8 | 51.026 |
| the aim of the present work is to | 13 | 0.002104 | 2 | 40.069 |
| the objective of this | 15 | 0.002428 | 5 | 38.141 |

Appendix 2.c

Description bundles in introduction

| Lexical bundles | Freq. in introduction | Normed occurrence | Freq. in RC | Keyness score |
| --- | --- | --- | --- | --- |
| the coordination chemistry of | 16 | 0.002595 | 1 | 55.771 |
| the development of a | 23 | 0.003723 | 17 | 41.224 |
| the development of new | 11 | 0.001781 | 1 | 36.781 |

Appendix 2.d

Literature-oriented bundles in introduction

| Lexical bundles | Freq. in introduction | Normed occurrence | Freq. in RC | Keyness score |
| --- | --- | --- | --- | --- |
| been the subject of | 27 | 0.004378 | 3 | 87.837 |
| have been extensively studied | 20 | 0.003237 | 1 | 71.105 |
| attention has been paid | 18 | 0.002913 | 0 | 70.961 |
| has been paid to | 18 | 0.002913 | 0 | 70.961 |
| attention in recent years | 14 | 0.002266 | 0 | 55.192 |
| has been extensively studied | 15 | 0.002428 | 2 | 47.419 |
| been devoted to the | 15 | 0.002428 | 2 | 47.419 |
| have been reported to | 23 | 0.003723 | 14 | 45.791 |
| have been devoted to | 13 | 0.002104 | 1 | 44.344 |
| has been the subject | 13 | 0.002104 | 2 | 40.069 |
| has been the subject of | 13 | 0.002104 | 2 | 40.069 |
| the subject of many | 13 | 0.002104 | 2 | 40.069 |
| attention has been paid to the | 10 | 0.001619 | 0 | 39.423 |
| been paid to the | 10 | 0.001619 | 0 | 39.423 |
| been focused on the | 10 | 0.001619 | 0 | 39.423 |
| much attention has been | 10 | 0.001619 | 0 | 39.423 |
| was paid to the | 10 | 0.001619 | 0 | 39.423 |
| have been widely studied | 10 | 0.001619 | 0 | 39.423 |
| have received considerable attention | 10 | 0.001619 | 0 | 39.423 |
| have also been reported | 17 | 0.002752 | 8 | 38.075 |
| have been the subject of | 11 | 0.00178 | 1 | 36.781 |
| have been the subject | 11 | 0.001789 | 1 | 36.781 |
| have focused on the | 11 | 0.001784 | 1 | 36.781 |
| have been shown to be | 13 | 0.002104 | 3 | 36.707 |

Appendix 2.e

Location bundles in introduction

| Lexical bundles | Freq. in introduction | Normed occurrence | Freq. in RC | Keyness score |
| --- | --- | --- | --- | --- |
| the last few years | 17 | 0.002752 | 0 | 67.019 |
| in the last few | 12 | 0.001942 | 0 | 47.307 |
| in the last few years | 12 | 0.001942 | 0 | 47.307 |

Appendix 2.f

Procedure bundles in introduction

| Lexical bundles | Freq. in introduction | Normed occurrence | Freq. in RC | Keyness score |
| --- | --- | --- | --- | --- |
| have been widely used | 22 | 0.003561 | 1 | 78.803 |
| the most widely used | 19 | 0.003075 | 3 | 58.278 |
| are widely used in | 16 | 0.002591 | 1 | 55.771 |
| have been developed for | 17 | 0.002752 | 3 | 51.012 |
| on the use of | 25 | 0.004047 | 16 | 48.511 |
| the most commonly used | 18 | 0.002913 | 5 | 48.376 |
| been widely used in | 15 | 0.002428 | 2 | 47.419 |
| have been used for | 21 | 0.003399 | 11 | 44.904 |
| have been used in | 17 | 0.002752 | 6 | 42.416 |
| has been widely used | 15 | 0.002428 | 4 | 40.777 |
| widely used in the | 12 | 0.001942 | 1 | 40.556 |
| been widely used as | 13 | 0.002104 | 2 | 40.069 |
| have been used to | 31 | 0.005018 | 37 | 39.573 |
| have also been used | 11 | 0.001781 | 1 | 36.781 |

Appendix 2.g

Structuring signals in introduction

| Lexical bundles | Freq. in introduction | Normed occurrence | Freq. in RC | Keyness score |
| --- | --- | --- | --- | --- |
| in this paper we | 125 | 0.020284 | 22 | 375.31 |
| in this work we | 62 | 0.010035 | 26 | 145.42 |
| this paper we report | 37 | 0.005989 | 1 | 136.92 |
| the present work we | 28 | 0.004532 | 9 | 72.034 |
| in this paper we report | 37 | 0.005989 | 1 | 136.92 |
| in the present work we | 28 | 0.004532 | 8 | 74.645 |
| paper we report the | 17 | 0.002752 | 0 | 67.019 |
| in this paper the | 34 | 0.005503 | 23 | 64.055 |
| this paper we present | 19 | 0.003075 | 2 | 62.294 |
| in this paper we present | 19 | 0.003075 | 2 | 62.294 |
| in the present work | 68 | 0.011026 | 115 | 61.094 |
| the present study we | 23 | 0.003723 | 7 | 60.176 |
| in the present paper | 23 | 0.003723 | 7 | 60.176 |
| in the present study we | 23 | 0.003723 | 7 | 60.176 |
| this paper we report the | 15 | 0.002428 | 0 | 59.134 |
| in this paper we report the | 15 | 0.002428 | 0 | 59.134 |
| of this work is to | 18 | 0.002913 | 2 | 58.558 |
| we report on the | 20 | 0.003237 | 6 | 58.419 |
| this work is to | 18 | 0.002913 | 3 | 54.636 |
| herein we report the | 18 | 0.002913 | 3 | 54.636 |
| of the present work | 24 | 0.003885 | 13 | 50.542 |
| of this paper is | 20 | 0.003237 | 7 | 50.042 |
| this paper is to | 16 | 0.002591 | 3 | 47.402 |
| this work we report | 12 | 0.001942 | 0 | 47.307 |
| of this study was | 25 | 0.004047 | 17 | 46.966 |
| of this work is | 19 | 0.003075 | 7 | 46.714 |
| this study was to | 24 | 0.003885 | 16 | 45.574 |
| we wish to report | 13 | 0.002104 | 1 | 44.344 |
| in this article we | 14 | 0.002266 | 2 | 43.735 |
| in this work we report | 11 | 0.00178 | 0 | 43.365 |
| paper we describe the | 11 | 0.00178 | 0 | 43.365 |
| in this paper we describe | 11 | 0.00178 | 0 | 43.365 |
| this paper we describe | 11 | 0.001781 | 0 | 43.365 |
| of this study was to | 23 | 0.003723 | 16 | 42.67 |
| of the present work is | 15 | 0.002428 | 4 | 40.777 |
| of the present work is to | 13 | 0.002104 | 2 | 40.069 |
| present work is to | 13 | 0.002104 | 2 | 40.069 |
| paper we report on | 10 | 0.001619 | 0 | 39.423 |
| the present paper is | 10 | 0.001619 | 0 | 39.423 |
| in this study we | 33 | 0.005341 | 45 | 37.318 |
| is to investigate the | 12 | 0.001942 | 2 | 36.424 |

Appendix 2.h

Framing signals in introduction

| Lexical bundles | Freq. in introduction | Normed occurrence | Freq. in RC | Keyness score |
| --- | --- | --- | --- | --- |
| in the treatment of | 33 | 0.005341 | 7 | 95.097 |
| in the field of | 35 | 0.005665 | 18 | 75.457 |
| for the treatment of | 31 | 0.005018 | 15 | 68.625 |
| for the development of | 27 | 0.004371 | 18 | 51.271 |
| in the study of | 20 | 0.003237 | 12 | 40.106 |
| for the detection of | 27 | 0.004375 | 28 | 38.614 |
| in the production of | 15 | 0.002428 | 5 | 38.141 |

Appendix 2.i

Stance features in introduction

| Lexical bundles | Freq. in introduction | Normed occurrence | Freq. in RC | Keyness score |
| --- | --- | --- | --- | --- |
| to the best of | 46 | 0.007446 | 29 | 89.961 |
| best of our knowledge | 44 | 0.007122 | 28 | 85.634 |
| to the best of our knowledge | 44 | 0.007122 | 28 | 85.634 |
| the best of our knowledge | 44 | 0.007122 | 28 | 85.634 |
| the best of our | 44 | 0.007122 | 29 | 84.066 |
| to the best of our | 44 | 0.007122 | 29 | 84.066 |
| an important role in | 44 | 0.007122 | 69 | 43.087 |
| play an important role | 32 | 0.005182 | 40 | 39.231 |
| there is a need | 12 | 0.001942 | 2 | 36.424 |

Appendix 3.a

Description bundles in results and discussion section

| Lexical bundles | Freq. in results and discussion | Normed occurrence | Freq. in RC | Keyness score |
| --- | --- | --- | --- | --- |
| the spectra of the | 75 | 0.003673 | 9 | 64.275 |
| the increase of the | 117 | 0.005731 | 29 | 63.593 |
| the intensity of the | 110 | 0.005388 | 26 | 62.264 |
| a function of the | 202 | 0.009894 | 82 | 61.662 |
| the values of the | 96 | 0.004702 | 29 | 43.028 |
| the ir spectra of | 69 | 0.003381 | 16 | 39.678 |
| the dihedral angle between | 32 | 0.001567 | 1 | 39.139 |
| the formation of the | 202 | 0.009894 | 106 | 38.002 |

Appendix 3.b

Structuring signal bundles in results and discussion section

| Lexical bundles | Freq. in results and discussion | Normed occurrence | Freq. in RC | Keyness score |
| --- | --- | --- | --- | --- |
| as shown in figure | 552 | 0.027037 | 91 | 402.32 |
| are shown in figure | 459 | 0.022482 | 59 | 381.28 |
| is shown in figure | 411 | 0.020131 | 90 | 247.36 |
| can be seen in | 156 | 0.007641 | 10 | 165.66 |
| be seen in figure | 97 | 0.004751 | 1 | 131.98 |
| can be seen in fig | 89 | 0.004359 | 1 | 120.46 |
| can be seen from | 103 | 0.005045 | 10 | 96.149 |
| as shown in table | 120 | 0.005878 | 17 | 95.035 |
| as can be seen in | 92 | 0.004506 | 7 | 93.128 |
| are shown in table | 136 | 0.006661 | 25 | 92.656 |
| are presented in figure | 84 | 0.004114 | 5 | 90.88 |
| are presented in table | 84 | 0.004114 | 14 | 60.822 |
| are listed in table | 163 | 0.007984 | 61 | 56.108 |
| are summarized in table | 120 | 0.005878 | 39 | 49.517 |
| as seen in figure | 46 | 0.002253 | 3 | 48.626 |
| shown in the inset | 35 | 0.001714 | 1 | 43.349 |
| be seen from figure | 32 | 0.001567 | 1 | 39.139 |
| are reported in table | 53 | 0.002596 | 9 | 37.948 |
| results are shown in | 57 | 0.002792 | 11 | 37.596 |
| be seen in table | 34 | 0.001665 | 2 | 36.891 |

Appendix 3.c

Resultative bundles in results and discussion section

| Lexical bundles | Freq. in results and discussion | Normed occurrence | Freq. in RC | Keyness score |
| --- | --- | --- | --- | --- |
| as a function of | 604 | 0.029584 | 277 | 149.92 |
| is due to the | 177 | 0.008669 | 45 | 94.063 |
| to the presence of | 160 | 0.035125 | 51 | 67.535 |
| as a function of the | 184 | 0.007837 | 73 | 58.178 |
| effect of ph on | 40 | 0.001959 | 3 | 40.664 |
| due to the presence | 100 | 0.004898 | 33 | 40.521 |
| the result of the | 117 | 0.005731 | 44 | 39.997 |
| is due to the presence of | 99 | 0.004849 | 33 | 39.631 |
| a result of the | 84 | 0.004114 | 25 | 38.257 |
| as a result the | 75 | 0.003673 | 21 | 36.376 |

Appendix 3.d

Framing signals in results and discussion section

| Lexical bundles | Freq. in results and discussion | Normed occurrence | Freq. in RC | Keyness score |
| --- | --- | --- | --- | --- |
| in the case of | 776 | 0.038008 | 313 | 239.29 |
| in the spectra of | 90 | 0.004408 | 7 | 90.5 |
| with the increase of | 113 | 0.005535 | 19 | 81.392 |
| in the case of the | 179 | 0.008767 | 62 | 68.321 |
| to the loss of | 76 | 0.003722 | 11 | 59.525 |
| to the formation of | 206 | 0.01009 | 94 | 51.6 |
| in the presence of | 763 | 0.037372 | 560 | 48.23 |
| in the spectrum of | 53 | 0.002596 | 6 | 46.575 |
| the increase in the | 76 | 0.003722 | 18 | 42.946 |
| the loss of the | 38 | 0.001861 | 2 | 42.306 |
| a decrease in the | 101 | 0.004947 | 33 | 41.415 |
| with respect to the | 308 | 0.015086 | 60 | 40.543 |
| in the ir spectra | 36 | 0.001763 | 2 | 39.592 |

Appendix 3.e

Transition signals in results and discussion section

| Lexical bundles | Freq. in result and discussion | Normed occurrence | Freq. in RC | Keyness score |
| --- | --- | --- | --- | --- |
| on the other hand | 570 | 0.027919 | 150 | 136.29 |

Appendix 3.f

Stance features in results and discussion section

| Lexical bundles | Freq. in results and discussion | Normed occurrence | Freq. in RC | Keyness score |
| --- | --- | --- | --- | --- |
| be attributed to the | 163 | 0.007984 | 28 | 115.9 |
| in agreement with the | 179 | 0.008767 | 37 | 112.49 |
| can be attributed to | 146 | 0.007151 | 25 | 104.01 |
| can be attributed to the | 100 | 0.004898 | 10 | 92.326 |
| is consistent with the | 131 | 0.006416 | 26 | 84.746 |
| be due to the | 152 | 0.007445 | 37 | 83.923 |
| is in agreement with | 119 | 0.005829 | 21 | 83.214 |
| the fact that the | 167 | 0.00818 | 47 | 80.586 |
| can be assigned to | 63 | 0.003086 | 2 | 76.878 |
| be explained by the | 97 | 0.004751 | 17 | 68.122 |
| higher than that of | 121 | 0.005927 | 29 | 67.696 |
| this indicates that the | 56 | 0.002743 | 2 | 67.107 |
| is in agreement with the | 76 | 0.003722 | 9 | 65.512 |
| may be due to | 116 | 0.005682 | 29 | 62.575 |
| it is clear that | 91 | 0.004457 | 17 | 61.345 |
| in good agreement with | 135 | 0.006612 | 42 | 58.576 |
| lower than that of | 69 | 0.00338 | 12 | 48.69 |
| can be explained by | 89 | 0.004359 | 22 | 48.489 |
| it is obvious that | 50 | 0.002449 | 5 | 46.163 |
| is assigned to the | 47 | 0.002302 | 4 | 45.931 |
| be assigned to the | 46 | 0.002253 | 4 | 44.634 |
| in good agreement with the | 78 | 0.003821 | 19 | 43.039 |
| it is interesting to | 66 | 0.003233 | 13 | 42.923 |
| is obvious that the | 29 | 0.00142 | 0 | 42.402 |
| it is obvious that the | 29 | 0.00142 | 0 | 42.402 |
| are similar to those | 47 | 0.002302 | 5 | 42.365 |
| is close to the | 41 | 0.002008 | 3 | 41.983 |
| might be due to | 34 | 0.001665 | 1 | 41.944 |
| be related to the | 67 | 0.003282 | 14 | 41.77 |
| is in good agreement | 46 | 0.002253 | 6 | 37.944 |
| may be attributed to | 59 | 0.00289 | 12 | 37.511 |
| are in agreement with | 63 | 0.003086 | 14 | 37.482 |
| is in good agreement with | 45 | 0.002204 | 6 | 36.73 |
| this is consistent with | 45 | 0.002204 | 6 | 36.73 |

Appendix 3.g

Engagement features in results and discussion section

| Lexical bundles | Freq. in results and discussion | Normed occurrence | Freq. in RC | Keyness score |
| --- | --- | --- | --- | --- |
| it can be seen | 259 | 0.012686 | 15 | 282.09 |
| as can be seen | 208 | 0.010188 | 14 | 218.04 |
| it can be seen that | 171 | 0.008376 | 10 | 185.81 |
| can be seen that | 171 | 0.008376 | 10 | 185.81 |
| be seen that the | 102 | 0.004996 | 7 | 106.35 |
| it can be seen that the | 93 | 0.004555 | 7 | 94.444 |
| it can be observed | 52 | 0.002547 | 2 | 61.549 |
| as it can be seen | 40 | 0.001959 | 0 | 58.485 |
| as can be seen from | 61 | 0.002988 | 6 | 56.668 |
| it should be noted | 109 | 0.005339 | 29 | 55.552 |
| as it can be | 47 | 0.002302 | 2 | 54.635 |
| should be noted that | 104 | 0.005094 | 27 | 54.226 |
| it should be noted that | 103 | 0.005045 | 27 | 53.228 |
| can be seen the | 36 | 0.001763 | 0 | 52.637 |
| can be observed that | 37 | 0.001812 | 2 | 40.948 |
| interesting to note that | 49 | 0.0024231 | 7 | 38.639 |
| it can be observed that | 35 | 0.001714 | 2 | 38.24 |
